# Programmable Base Editing in *Mycobacterium tuberculosis* Using an Engineered CRISPR RNA-Guided Cytidine Deaminase

**DOI:** 10.3389/fgeed.2021.734436

**Published:** 2021-12-08

**Authors:** Xin-Yuan Ding, Si-Shang Li, Yi-Man Geng, Mei-Yi Yan, Guo-Bao Li, Guo-Liang Zhang, Yi-Cheng Sun

**Affiliations:** ^1^ NHC Key Laboratory of Systems Biology of Pathogens, Institute of Pathogen Biology, Center for Tuberculosis Research, Chinese Academy of Medical Sciences and Peking Union Medical College, Beijing, China; ^2^ Department of Clinical Laboratory, Henan Provincial People’s Hospital, People’s Hospital of Zhengzhou University, Zhengzhou, China; ^3^ National Clinical Research Center for Infectious Diseases, Shenzhen Third People’s Hospital, Southern University of Science and Technology, Shenzhen, China

**Keywords:** mycobacteria, cytidine deaminase, genome editing, CRISPR, base editing

## Abstract

Multidrug-resistant *Mycobacterium tuberculosis* (*Mtb*) infection seriously endangers global human health, creating an urgent need for new treatment strategies. Efficient genome editing tools can facilitate identification of key genes and pathways involved in bacterial physiology, pathogenesis, and drug resistance mechanisms, and thus contribute to the development of novel treatments for drug-resistant *tuberculosis*. Here, we report a two-plasmid system, *MtbCBE*, used to inactivate genes and introduce point mutations in *Mtb*. In this system, the assistant plasmid pRecX-NucS_E107A_ expresses RecX and NucS_E107A_ to repress RecA-dependent and NucS-dependent DNA repair systems, and the base editor plasmid pCBE expresses a fusion protein combining cytidine deaminase APOBEC1, Cas9 nickase (nCas9), and uracil DNA glycosylase inhibitor (UGI). Together, the two plasmids enabled efficient G:C to A:T base pair conversion at desired sites in the *Mtb* genome. The successful development of a base editing system will facilitate elucidation of the molecular mechanisms underlying *Mtb* pathogenesis and drug resistance and provide critical inspiration for the development of base editing tools in other microbes.

## Introduction

*Mycobacterium tuberculosis* (*Mtb*) is the causative agent of *tuberculosis* (TB) and the leading cause of death from a single pathogen. The World Health Organization (WHO) estimated that, in 2019, 10 million new patients around the world were diagnosed with TB (World Health Organization, 2020). The emergence of multidrug-resistant *tuberculosis* (MDR-TB) and extensively drug-resistant *tuberculosis* (XDR-TB) strains is an ongoing health problem that creates an urgent need for novel therapeutic strategies. Identification and characterization of drug targets strongly rely on efficient genetic manipulation techniques. However, genetic manipulation of mycobacteria is challenging, mainly due to their slow growth, the pathogenicity of some species, and their GC-rich genomes.

Recently, several techniques for genetic engineering have been developed in mycobacteria (Chhotaray et al., 2018; Murphy et al., 2018), benefiting especially from the advent of the CRISPR-Cas system (Rock et al., 2017; Yan et al., 2017; Yan et al., 2020). We have reported a highly efficient system based on recombineering and CRISPR-Cas12a-mediated counterselection (Yan et al., 2017), that can rapidly generate point mutations, deletions, and insertions in *Mycobacterium smegmatis*. In addition, we developed a CRISPR-Cas-mediated NHEJ genome editing tool that allowed us to generate markerless deletions in *M. smegmatis* and *Mtb* (Yan et al., 2020). However, none of them could be used to introduce point mutations or to perform accurate genetic manipulation in *Mtb*.

Fortunately, the discovery of single-base editors provides a novel strategy for precise genetic manipulation without the need to introduce double-strand breaks (DSBs) or donor DNA templates (Komor et al., 2016; Gaudelli et al., 2017). The cytidine base editor (CBE) consists of a catalytically impaired Cas nuclease alongside a cytidine deaminase (such as APOBEC) and the uracil DNA glycosylase inhibitor UGI, which catalyzes a conversion of cytosine (C) to uracil (U) at targeted sites, resulting in substitution of C to thymine (T) (Komor et al., 2017). Base editing systems have been extended to microorganisms, such as *Escherichia coli* (Banno et al., 2018; Zheng et al., 2018), *Staphylococcus aureus* (Gu et al., 2018), *Klebsiella pneumoniae* (Wang et al., 2018), and *Streptomyces* (Tong et al., 2019; Zhao et al., 2020), greatly promote the genetic engineering of these bacteria.

In this study, we constructed a two-plasmid cytidine base editing system (*MtbCBE*) for genome editing in *Mtb*. In this system, pRecX-NucS_E107A_ encodes RecX and NucS_E107A_ to repress homologous recombination (HR) and mismatch repair (MMR) DNA repair pathway, respectively, and together with pCBE encodes a codon-optimized fusion of nCas9, cytidine deaminase APOBEC1, and UGI to generate G:C to A:T base pair conversion at desired sites in *Mtb*. The development of a CBE system for *Mtb* holds great promise for the study of mycobacterial physiology and should aid in the development of anti-tuberculosis drugs.

## Materials and Methods

### Strains, Media, and Growth Conditions

*Mtb* strains H37Ra, *M. smegmatis* strain mc^2^ 155, and their derivatives were used in this study; all strains are listed in Supplementary Table S1. Mycobacteria were grown in Middlebrook 7H9 broth (Difco) supplemented with 0.05% Tween 80 and 0.2% glycerol or on Middlebrook 7H10 plates. Additional oleic acid-albumin-dextrose-catalase (OADC) was required for *Mtb*. Appropriate antibiotics were supplemented as necessary: kanamycin, 25 μg/ml; bleomycin, 50 μg/ml.

### Mutant Construction

*nucS* deletion strains were constructed using a CRISPR/Cas-assisted recombineering method (Yan et al., 2017). Briefly, dsDNA fragments containing in-frame deletions in the *nucS* region were constructed and co-transformed with a crRNA plasmid targeting *nucS*. The electroporated cells were recovered in 7H9 broth and then plated on 7H10 agar containing 50 ng/ml anhydrotetracycline (ATc) and appropriate antibiotics. Mutant colonies were confirmed by PCR and sequencing.

### Plasmid Construction

The plasmids used in this study are listed in Supplementary Table S1. To construct pCBE, deaminase and UGI were codon-optimized (Supplementary Table S3) and cloned by CRISPR-Cas12a and phage λ Red recombineering (Geng et al., 2019) into a plasmid containing dCas9_sth1_/nCas9_sth1_ and the cognate sgRNA scaffold. APOBEC1 (lacking a stop codon) was fused to the N-terminus of nCas9_sth1_/dCas9_sth1_ (lacking start and stop codons) *via* 16-amino acid flexible linker. UGI (lacking a start codon) was then fused to the C-terminus of dCas9_sth1_/nCas9_sth1_ *via* a Ser-Gly-Gly-Ser linker. *sacB*-*oriM* was amplified by PCR from pSL003 and cloned into pCBE digested with *Xba*I and *Nhe*I. The annealed sgRNA spacer oligonucleotides were inserted into the *Bsm*BI sites of pCBE by Golden Gate assembly. sgRNA spacers are listed in Supplementary Table S2. To construct pRecX, *recX* was amplified by PCR from *M. smegmatis* and cloned into pMV261; Km^r^ was replaced with Zeo^r^. To create a dominant-negative mutant of *nucS*, various *nucS* point mutants were commercially synthesized and cloned into pMV261 digested with *Pst*I and *Hind*III. To construct pRecX-NucS_E107A_, *recX*, and *nucS*_E107A_ were introduced into a plasmid by seamless cloning.

### Base Editing Assay

For base editing in mycobacteria, 200–300 ng of editor plasmid was transformed into competent cells by electroporation (2.5 kV, 1,000 Ω, 25 μF), and the cells were recovered for 4 h (24 h for *Mtb*) in 1 ml of 7H9 medium at 37°C. Samples were plated on 7H10 agar supplemented with appropriate antibiotics and 50 ng/ml ATc. After growth for 4 days (28–30 days for *Mtb*) at 37°C, colonies on plates were counted or examined for loss of the GFP signal by fluorescence excitation flashlights if necessary, and then by PCR and DNA sequencing. The editing efficiency was calculated by the ratio of successfully edited colonies to the total numbers of randomly picked colonies. Primers used for PCR are listed in Supplementary Table S2.

### Estimation of Mutation Rates

Mutation rates was estimated by fluctuation analysis. For each experiment, 10 cultures (2 ml of 7H9) from single colonies were grown to saturation at 30°C. Saturated cultures were diluted 1:100 in 3 ml 7H9 medium and incubated for 24 h. Appropriate dilutions of the cultures were plated on 7H10 plates with or without 100 μg/ml rifampicin. The mutation rate was calculated as described using the Salvador web tool (https://websalvador.eeeeeric.com/) (Castaneda-Garcia et al., 2017).

### MIC_50_ Determination

The 50% minimum inhibitory concentration (MIC_50_) of wild-type H37Ra and *katG* mutant was determined by the broth microdilution method in 96-well plates. INH drug solution was serially 2-fold diluted and added to 96-well clear plates (0.1 ml per well). A volume of 0.1 ml of a logarithmic phase *Mtb* culture (diluted to OD_600_ of 0.04) was added to each well, and the plates were incubated at 37°C for 5 days. OD_600_ was measured using a spectrophotometer, and MIC_50_ curves were plotted using the GraphPad Prism 8.3 software.

## Results

### Cytidine Base Editing in *M. smegmatis*

**FIGURE 1:**
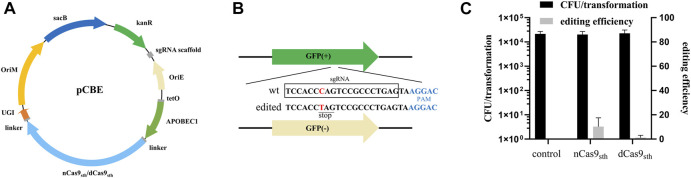
Cytidine base editing in *M. smegmatis*. **(A)** Map of plasmid pCBE. The single editor plasmid contains: KanR, kanamycin-resistance marker; OriE, origin of replication in *E. coli*; OriM, origin of replication in mycobacteria; sgRNA cassette; a fusion protein expression cassette including dCas9_sth1_ or nCas9_sth1_ with APOBEC1 at the N-terminus and UGI at the C-terminus, under the control of the ATc-inducible P*tetO* promoter; and the *sacB* gene for plasmid curing. **(B)** *gfp*-targeting sequence. Successful editing introduces a TAG stop codon, resulting in loss of green fluorescence. **(C)** Colony-forming units (CFU) per transformation and editing efficiency of APOBEC1-nCas9_sth1_/dCas9_sth1_-UGI. Editing efficiency was calculated based on the proportion of GFP-negative colonies. Results are the average of at least two independent experiments, and error bars depict the standard deviations.

To determine whether deaminase-mediated targeted mutagenesis could be achieved in *M. smegmatis*, we constructed base editor plasmids (pCBEs) expressing a fusion protein consisting of dead Cas9 (dCas9) or nCas9 from *Streptococcus* thermophilus (Rock et al., 2017), cytidine deaminase (APOBEC1) at the N-terminus, and UGI at the C-terminus under the control of the anhydrotetracycline (ATc)-inducible P*tetO* promoter; the plasmids also expressed an sgRNA cassette under the control of a constitutive promoter (Figure 1A). To test the editing efficiency of this CBE system, we took advantage of the genetically modified *M. smegmatis* strain MC^2^ 155 strain, which harbors green fluorescent protein (GFP) reporter gene as a readily screened target for genome editing (Yan et al., 2017). We designed an sgRNA in which the target C was at position 7, counting the PAM as positions 23–27; this sequence should direct C-to-T conversion at Gln204, introducing a premature TAG stop codon, resulting in loss of the GFP signal (Figure 1B). Editing efficiency was calculated based on the proportion of GFP-negative colonies on plates. As shown in Figure 1C, transformation with the CBE plasmid with targeting sgRNA (pCBE-*gfp*) yielded ∼10^4^ colonies, of which 10.3% were GFP-negative when APOBEC1-nCas9_sth1_-UGI fusion was used. By contrast, the editing efficiency of APOBEC1-dCas9_sth1_-UGI was only 1.2% (Figure 1C). These data indicate that APOBEC1-nCas9_sth1_-UGI might work better than APOBEC1-dCas9_sth1_-UGI for base editing in *M. smegmatis*.

### RecX Affects the Efficiency of Single Base Editing

**FIGURE 2:**
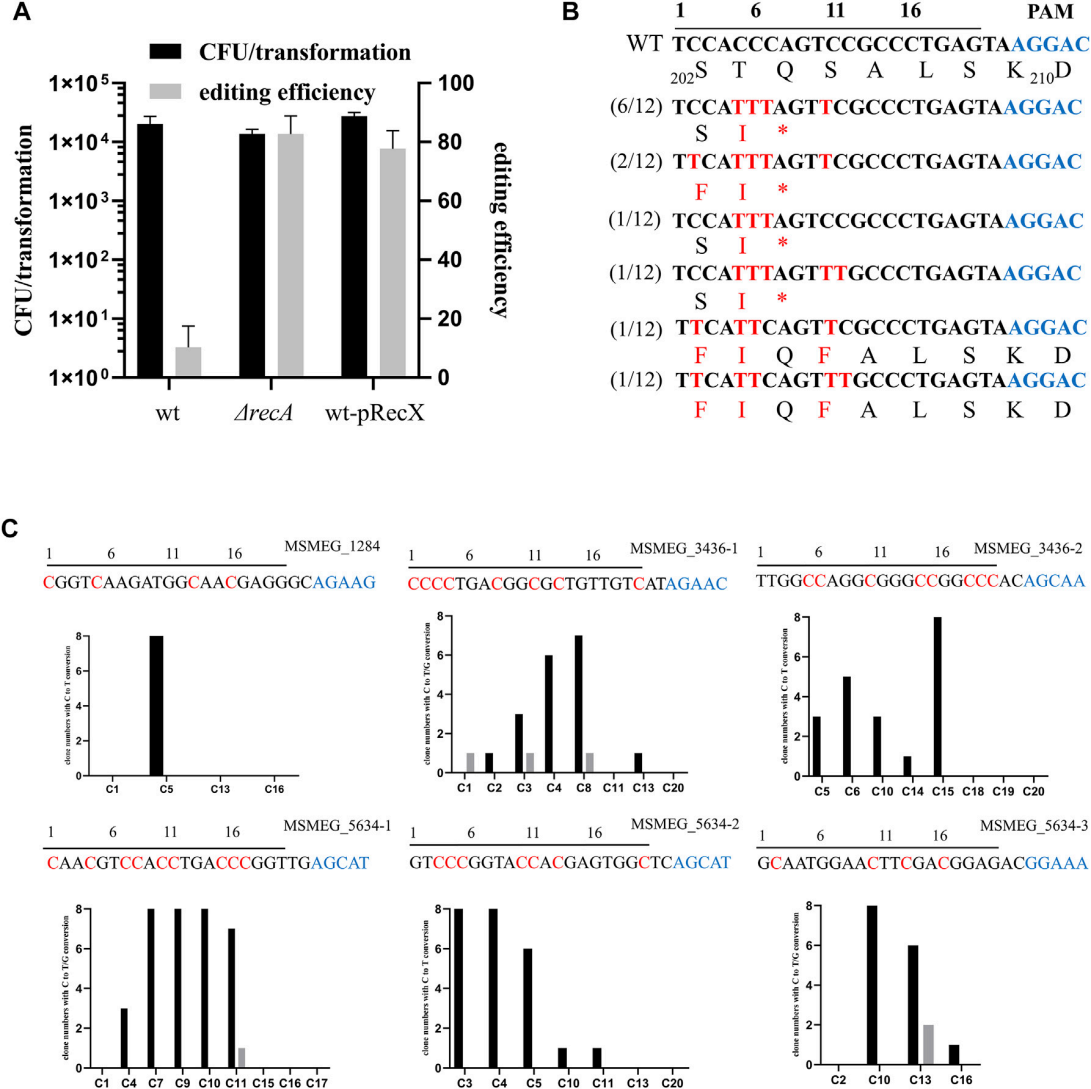
RecX affects the efficiency of single base editing. **(A)** *recA* deletion and *recX* overexpression increase the editing efficiency of CBE. **(B)** Sequence alignments of the targeted loci. Black line over the alignment indicates *gfp* sgRNA and PAM motif is shown in blue. The targeted sites from 12 randomly-picked clones edited by APOBEC1-nCas9_sth1_-UGI were sequenced and aligned. The mutated bases and amino acids are shown in red. **(C)** Examination of the editing window of the CBE system in *M. smegmatis*. Six spacers were assembled into the pCBE plasmid, respectively. The resulted plasmids were transformed into the *M. smegmatis* strain. Eight randomly-picked clones from each target were sequenced and the number of clones with edited C at different positions were counted. C to T conversion is colored black and C to G conversion is colored grey. Black line over the alignment indicates sgRNA spacers and PAM motif is shown in blue. The expected editing Cs are indicated by red.

nCas9_sth1_ still retains nickase activity, which can nick the non-edited strand of the DNA, and a single-strand break is capable of initiating the HR DNA repair pathway (Komor et al., 2016). Hence, we hypothesized that mutation of *recA*, the key player in the HR pathway, has the potential to increase base editing efficiency. To test this, we transformed the nCas9_sth1_ editor described above into a *recA* null strain and performed the *gfp* gene-editing assay. As shown in Figure 2A, in the absence of *recA*, editing efficiency increased to 82%, indicating that loss of the RecA-mediated HR pathway could increase base editing efficiency.

To facilitate base editing in various strain backgrounds, we sought to construct a system that could limit RecA function without disrupting the *recA* gene. Because overexpression of RecX represses RecA activity (Yan et al., 2020), we constructed a new two-plasmid CBE system based on RecX. Plasmid pRecX, expressing *M. smegmatis recX*, was electroporated into wild-type cells to obtain a *recX*-harboring strain, which was collected and prepared as competent cells and then transformed with pCBE-*gfp*. As expected, expression of *recX* yielded editing efficiency similar to that of *recA* deletion, with 78% edited transformants (Figure 2A).

We randomly picked 12 GFP-negative colonies for PCR and sequencing. Almost all Cs at positions 5–11 could be edited. The cytidines at positions 2 and 12 were also mutated, albeit with a lower frequency (4/12 and 2/12, respectively). Exceptionally, in two strains, the cytidine at position 7 was not edited and, consequently, no premature TAG stop codon was generated (Figure 2B). However, these GFP negative colonies remained negative when they were streaked on plates without Atc suggesting the loss of GFP signal was not due to potential regulatory effects of nCas9_sth_. In addition, 4 GFP-positive colonies were picked for sequencing and confirmed that their GFP encoding gene has not been edited. In this case, the lack of a GFP signal may have been due to multiple mutations in adjacent amino acids.

To further investigate the editing window of our CBE system in mycobacteria, we designed six sgRNA spacers containing Cs at different positions to examine the editing efficiency. As shown in Figure 2C, the conversion efficiency of C to T varies in different positions. The C at positions 1 and 2 can hardly be mutated whereas from positions 3 to 9, the editing efficiency has increased significantly, ranging from 37.5 to 100%. Editing events as far as positions 10 and 11 were also observed and C after position 11 can barely be edited (). In addition, we found that a few Cs were mutated to guanines (Gs) (Figure 2C, grey), which was also observed in base editing of mammalian cells (Ma et al., 2016; Nishida et al., 2016).

### NucS Affects the Efficiency of Single-Base Editing

**FIGURE 3:**
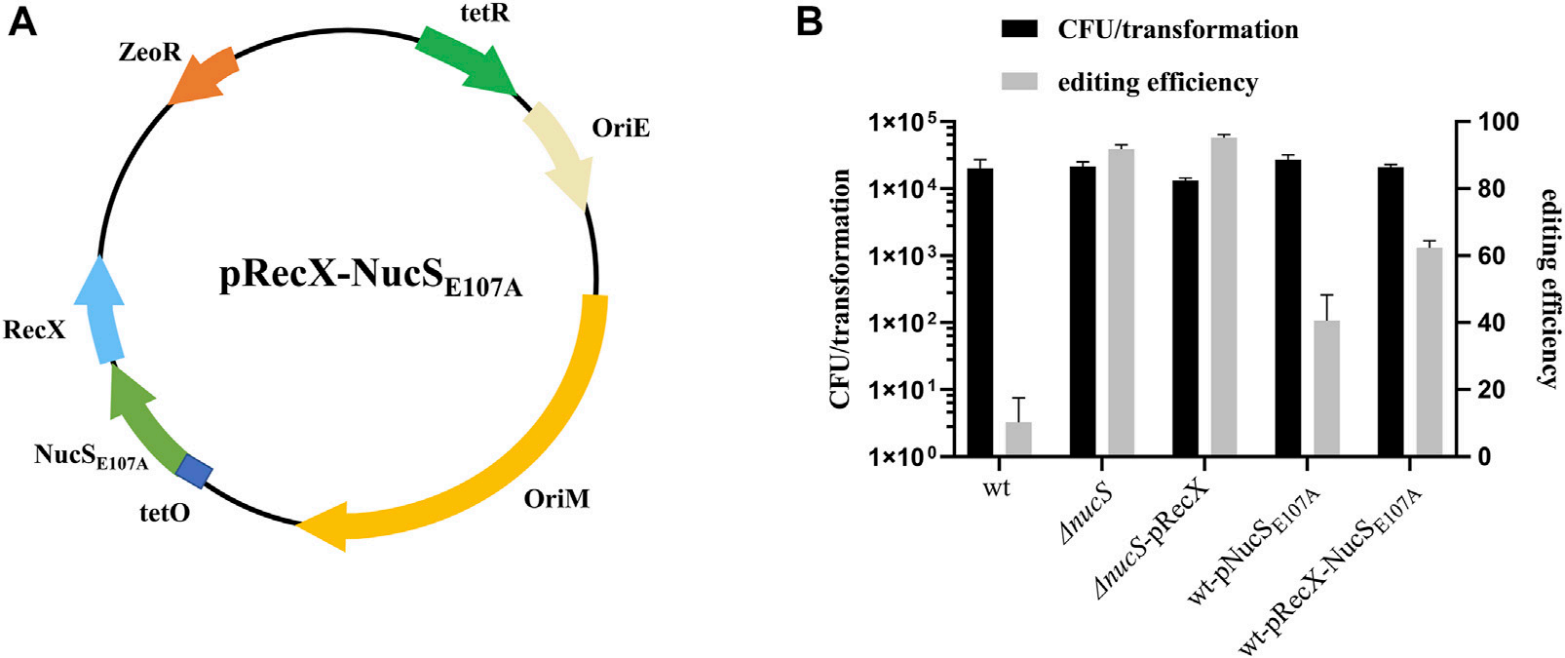
NucS affects the efficiency of single base editing. **(A)** Map of plasmid pRecX-NucS_E107A_. The plasmid contains: ZeoR, bleomycin-resistance marker; OriE and OriM; RecX and NucS_E107A_ under the control of the P*tetO* promoter; and tetR, tetracycline repressor. **(B)** Deletion of *nucS* significantly increased base editing efficiency, and NucS_E107A_ had a dominant-negative effect.

MMR is a highly conserved DNA repair process that corrects mismatched nucleotide base pairs during DNA replication. Recent work showed that NucS has no structural homology with known MMR factors but still has hallmarks of canonical MMR in *M. smegmatis* (Castaneda-Garcia et al., 2017; Castañeda-García et al., 2020). NucS endonuclease from Thermococcus gammatolerans is capable of cleaving U- and inosine (I)-containing double-strand DNA (dsDNA), suggesting that NucS might be involved in repair of deaminated bases (Zhang et al., 2020). To determine whether NucS in *M. smegmatis* could affect the efficiency of base editing, we constructed a *nucS* knockout strain and transformed pCBE-*gfp* into this mutant. The editing efficiency increased substantially to 92%, in the *nucS* mutant (Figure 3B). Moreover, we achieved 95% editing efficiency when we combined the *nucS* mutant with overexpression of *recX* (Figure 3B). These results suggest that mutation of *nucS* can strongly increase base editing efficiency in *M. smegmatis*.

To facilitate base editing in strains of various genetic backgrounds, we sought to inhibit NucS activity by expression of a mutant dominant-negative form of the protein. The NucS-S39R mutation, which has been reported in some *Mtb* clinical strains, might decrease NucS activity (Castaneda-Garcia et al., 2017). Trp52 is in the highly conserved N-terminal DNA-binding domain, whereas Glu107 and Asp140 are in the conserved catalytic active site in the C-terminal RecB-like nuclease domain (Supplementary Figure S1) (Ren et al., 2009). Hence, we constructed the S39R, W52A, E107A, and D140A *nucS*_Mtb_ mutants in the shuttle expression plasmid pMV261 and transformed the resultant plasmids into *M. smegmatis* to test their effects on NucS activity by monitoring the frequency of spontaneous rifampicin resistance. Notably, NucS_E107A_ exerted a slight dominant-negative effect (Table 1, fold change = 1.72), suggesting that overexpression of this mutant inhibited native NucS-mediated MMR. Hence, we next tested whether NucS_E107A_ could improve base editing efficiency. Introduction of NucS_E107A_ slightly increased editing efficiency (Figure 3B), partially mimicking the phenotype of *nucS* mutant. However, plasmid pRecX-NucS_E107A_ (Figure 3A) co-expressing RecX and NucS_E107A_ was not more efficient than expressing either alone (Figure 3B).

**TABLE 1:** Mutation rates of *M. smegmatis* and its *nucS* mutant allele derivatives. Rates of spontaneous mutations conferring rifampicin resistance in *M. smegmatis* mc^2^ 155 (WT) and wild-type host expressing a mutant allele of *nucS*.

*nucS* derivatives	wt	S39R	W52A	E107A	D140A	Δ*nucS*	*ΔnucS::nucS*
Mutation rate (×10^−8^)	1.51	1.57	1.14	2.59	0.93	32.1	1.93
95%CI (×10^−8^)	0.83–2.47	0.87–2.43	0.60–1.86	1.50–4.08	0.44–1.69	23.1–40.8	0.99–3.20

### Cytidine Base Editing in *Mtb*

**FIGURE 4:**
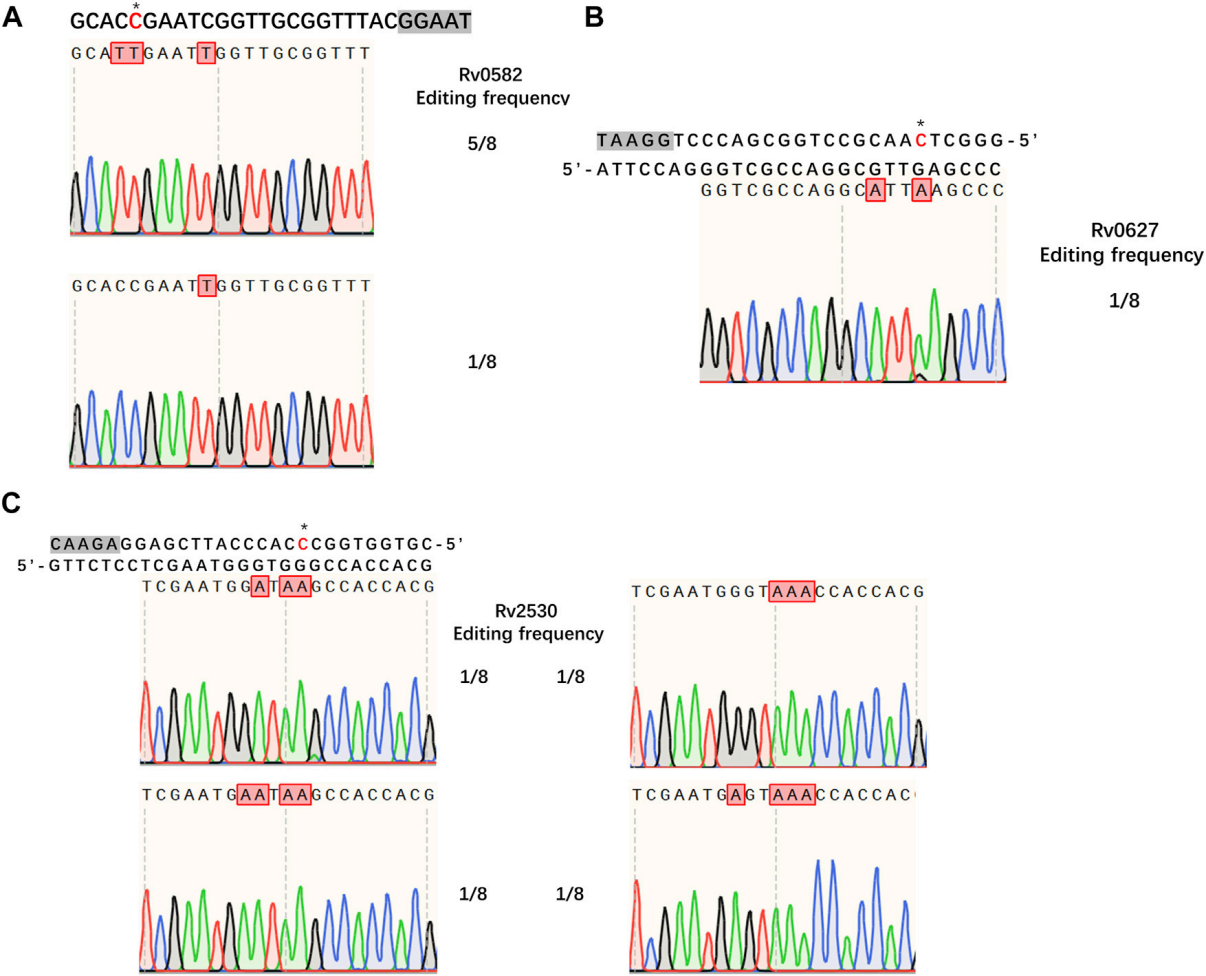
Cytidine base editing in *Mtb.* **(A–C)** Sequence alignments of the targeted loci from eight randomly picked colonies: **(A)** *Rv0582*, **(B)** *Rv0627*, **(C)** *Rv2530*. A grey box indicates the PAM sequence. Desired cytidines are marked with an asterisk and edited cytidines are shown in red.

Having demonstrated the successful establishment of an effective CBE system in *M. smegmatis*, our next goal was to evaluate whether the system would work in *Mtb*. For editing, we chose three genes encoding toxins of the *Mtb* toxin-antitoxin (TA) system, *Rv0582* (*Ra0589*), *Rv0627* (*Ra0636*), and *Rv2530* (*Ra2557*). The sgRNA sequences of these genes were designed to introduce premature stop codons, thus inactivating the corresponding genes. We first tested the functionality of our system in *Mtb* strain H37Ra. To our surprise, no detectable base editing was detected when pCBE with targeting sgRNA was transformed into cells, with or without pRecX (data not shown), whereas editing efficiencies of 75, 12.5, and 50% for the three targeted genes were achieved when pCBE was transformed into cells harboring pRecX-NucS_E107A_ (Figure 4).

**FIGURE 5:**
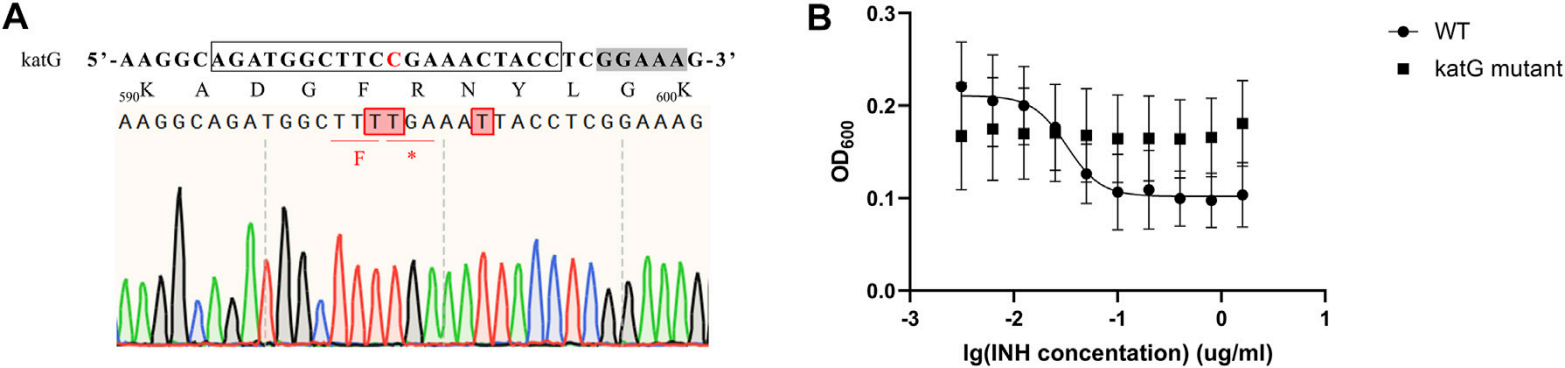
*katG* inactivation confers high-level resistance to INH. **(A)** Edited *katG* gene loci were analyzed by deep sequencing. sgRNA sequence is boxed and a grey box indicates the PAM sequence. Edited cytidines and amino acids are highlighted in red. **(B)** MIC_50_ values of wild-type H37Ra and *katG* mutant. These experiments were carried out in triplicate. Data are given as mean values and standard deviations.

Isoniazid (INH) is one of the most important first-line drugs used in anti-TB regimens, and the emergence of drug-resistant *Mtb* strains is increasing worldwide. Resistance to INH is commonly due to non-sence point mutations in the *Rv 1908c* gene, which encodes the catalase-peroxidase KatG enzyme and is indispensable for activation of INH. To confirm that our *MtbCBE* system could be used to characterize drug resistance genes, we used this method to inactivate *katG* and assessed the INH susceptibility of the wild-type and mutant strains. Sequencing analysis revealed that one of the eight selected transformants was mutated at the desired position (11, counting the PAM as positions 23–27); cytidines at position 10 and 16 were also mutated (Figure 5A). Consistent with previous reports, the resultant mutant had a higher 50% minimum inhibitory concentration (MIC_50_) (>1.6 μg/ml) than the wild-type strain (MIC_50_ ≈ 0.034 μg/ml) (Figure 5B). Taken together, these results demonstrated that the *MtbCBE* system can efficiently generate G:C-to-A:T base pair conversion in *Mtb* and can be effective in functional characterization of genes.

### Discussion

As the prevalence of antibiotic-resistant *Mtb* continues to rise, development of novel anti-tuberculosis drugs becomes increasingly urgent. However, the difficulties of genetic manipulation of this species have greatly hindered TB research. Recently, deaminase-mediated base editing technology has attracted a great deal of attention as a genome-engineering tool. Here, we demonstrate that the CBE system could be used to achieve base editing in *Mtb* if the DNA repair system was repressed in parallel. Distinct from CRISPR/Cas-assisted HR and NHEJ genetic tools, CRISPR/Cas-base editing is an alternative strategy that does not generate DSBs, thus rendering precise editing in *Mtb*. CRISPR interference (CRISPRi), developed for transcriptional inhibition in mycobacteria (Choudhary et al., 2015; Rock et al., 2017), has been recently used for genome-wide gene expression tuning (Bosch et al., 2021). However, this method has several limitations, such as polar effect and reduced efficiency due to negative feedback. By contrast, CRISPR/Cas9-base editing generates gene knockouts via premature stop codon rather than transcriptional knockdown, which greatly avoids the above problems.

In our CBE system, dCas9_sth1_ was less efficient than nCas9_sth1_under detected conditions. This is consistent with other results obtained using the base editor BE3 in mammalian cells (Komor et al., 2016) and *E. coli* (Zheng et al., 2018), but in contrast to those obtained using target-AID developed by Akihiko Kondo (Banno et al., 2018). More experiments involving varying PAM motifs and sgRNA spacer lengths are needed to support this observation. The DNA repair systems in mycobacteria, especially in *Mtb*, may be very powerful, causing base editing efficiency to be quite low (Figure 1C). This is supported by the observation that base editing efficiency could be improved by repression of RecA-dependent HR and NucS dependent MMR (Figures 2A, 3B).

In summary, we developed a cytidine base editor system that can efficiently generate C-to-T mutations in mycobacteria. Our *MtbCBE* system has several important applications in *Mtb*, including identification of key genes and pathways involved in bacterial physiology and facilitating the development of novel treatments for drug-resistant TB. In addition, the general approach of repressing DNA repair systems to increase base editing efficiency could be used for the development of base editing tools in other organisms.

## Data Availability

The datasets presented in this study can be found in online repositories. The names of the repository/repositories and accession number(s) can be found in the article/Supplementary Material.
