# Supplementary material for: Programmable Base Editing in Mycobacterium tuberculosis Using an Engineered CRISPR RNA-Guided Cytidine Deaminase
Source: Front Genome Ed. 2021 Dec 8;3:734436. doi: 10.3389/fgeed.2021.734436 (PMC8692370; doi:10.3389/fgeed.2021.734436)
Supplement: Supplementary file 1 [file DataSheet1.docx]

Supplementary Material

# Supplementary Figures and Tables

## Supplementary Figures


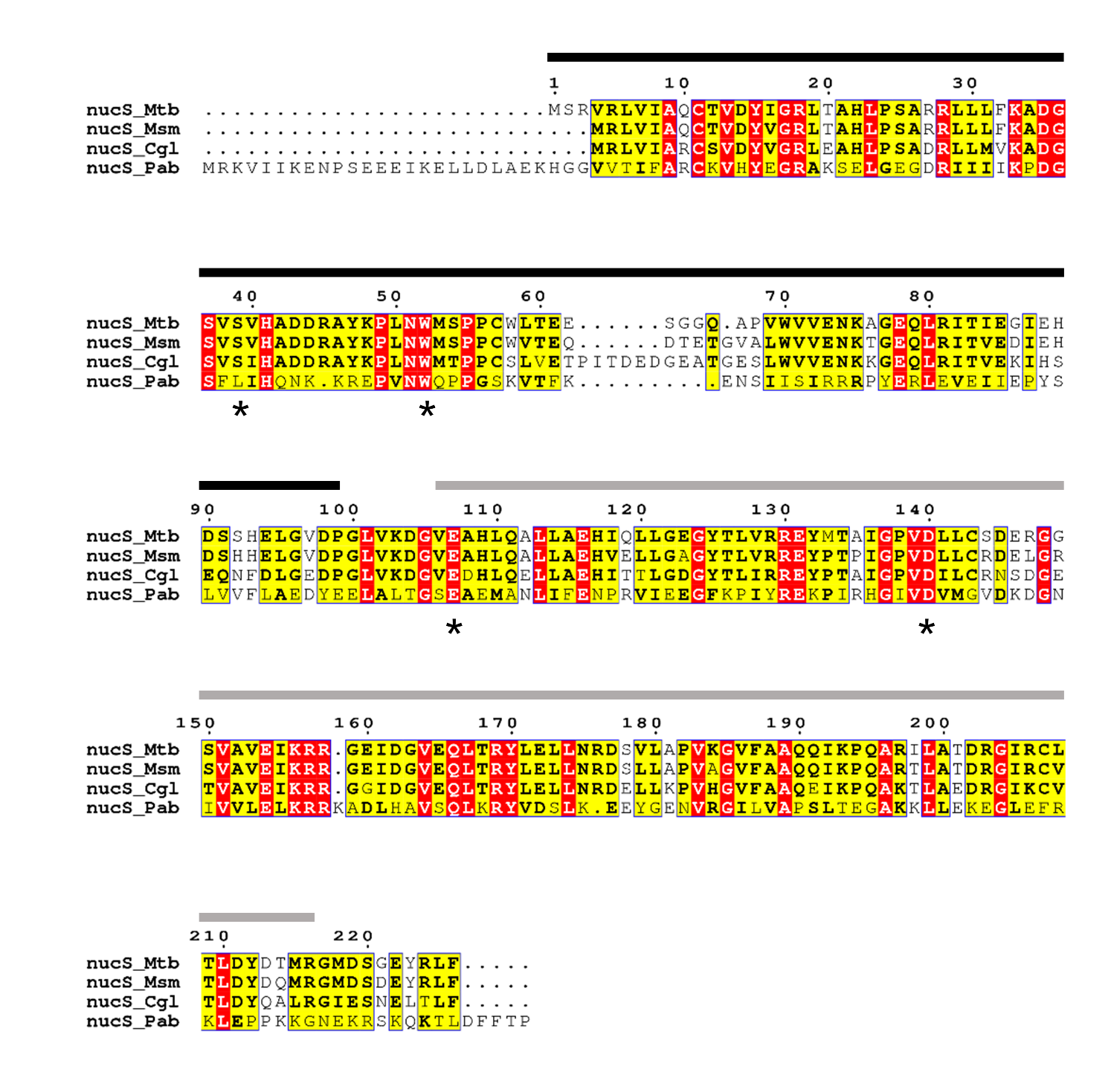


**Supplementary Figure 1.** Multiple amino-acid sequence alignment of NucS orthologs. Solid lines over the alignment indicate protein domains (black, N-terminal DNA-binding domain; gray, C-terminal RecB-like nuclease domain). The homologous regions of the sequences are boxed, and conserved residues are in red. The positions tested in this study are labeled with asterisks. *Mtb* (NucS_Mtb), *M. smegmatis* (NucS_Msm), *Corynebacterium glutamicum* (NucS_Cgl), and *Pyrococcus abyssi* (NucS_Pab).

## Supplementary tables

**Supplementary Table 1. Strains and plasmids used in this study**

| **Strains or plasmids** | **description** | **reference** |
| --- | --- | --- |
| **Strains** |  |  |
| *E. coli* |  |  |
| DH5α | General-purpose cloning strain | Lab stock |
| *M. smegmatis* |  |  |
| mc^2^155 | Wild-type *M. smegmatis* | Lab stock |
| SY4064 | *MS5634-5635* was replaced by *gfp* gene in mc^2^155 chromosome | reference (1) |
| SY4081 | Deletion of *recA* in mc^2^155 | reference (2) |
| SY6133 | SY4064 harboring *recX* from *M. smegmatis* | This study |
| *M. tuberculosis* |  |  |
| H37Ra | Wild-type *M. tuberculosis* | Lab stock |
| **Plasmids** |  |  |
| pCBE-dCas9 | CBE vector (dCas9_sth1_, sgRNA, APOBEC1, UGI, sacB) | This study |
| pCBE-nCas9 | dCas9_sth1_(A599) reverted to nCas9_sth1_ (H599) in pCBE-dCas9 | This study |
| pCBE-dCas9-*gfp* | pCBE-dCas9 with *gfp* spacer, Gln204 to stop codon | This study |
| pCBE-nCas9-*gfp* | pCBE-nCas9 with *gfp* spacer, Gln204 to stop codon | This study |
| pRecX | The *recX* from *M. smegmatis* inserted into pMV261 with zeo^r^ | This study |
| pRecX-NucS_E107A_ | Assistant plasmid for *Mtb*CBE | This study |
| pCBE-1284 | pCBE-nCas9 with *MSEMG_1284* spacer | This study |
| pCBE-3436-1 | pCBE-nCas9 with *MSEMG_3436* spacer 1 | This study |
| pCBE-3436-2 | pCBE-nCas9 with *MSEMG_3436* spacer 2 | This study |
| pCBE-5634-1 | pCBE-nCas9 with *MSEMG_5634* spacer 1 | This study |
| pCBE-5634-2 | pCBE-nCas9 with *MSEMG_5634* spacer 2 | This study |
| pCBE-5634-3 | pCBE-nCas9 with *MSEMG_5634* spacer 3 | This study |
| pCBE-0582 | pCBE-nCas9 with *Rv0582* spacer, Arg84 to stop codon | This study |
| pCBE-0627 | pCBE-nCas9 with *Rv0627* spacer, Gln62 to stop codon | This study |
| pCBE-2530 | pCBE-nCas9 with *Rv2630* spacer, Trp36 to stop codon | This study |
| pJV53-Cas12a | For CRISPR-based recombineering (*oriM*; *oriE*; Km^r^; Che9c; Cas12a) | reference (1) |
| pCR-Hyg | For CRISPR-based recombineering containing crRNA Cassette (pBR322 ori; pAL5000ts; Hyg^r^) | reference (1) |
| pMV261 | Shuttle vector; replicates extrachomosomally in both *E. coli* and mycobacterium (*oriM*; *oriE*; Km^r^) | reference (3) |

**Supplementary Table 2. Primers and sgRNA spacers used in this study**

| **name** | **sequence** |
| --- | --- |
| sgRNA-gfp | TCCACCCAGTCCGCCCTGAGTA |
| sgRNA-1284 | CGGTCAAGATGGCAACGAGG |
| sgRNA-3436-1 | CCCCTGACGGCGCTGTTGTC |
| sgRNA-3436-2 | TTGGCCAGGCGGGCCGGCCC |
| sgRNA-5634-1 | CAACGTCCACCTGACCCGGT |
| sgRNA-5634-2 | GTCCCGGTACCACGAGTGGC |
| sgRNA-5634-3 | GCAATGGAACTTCGACGGAG |
| sgRNA-0582 | gcaccgaatcggttgcggttt |
| sgRNA-0627 | gggctcaacgcctggcgacc |
| sgRNA-2530 | gtggtggcccacccattcga |
| F for GFP | ctacggcaagctgaccctga |
| R for GFP | tttaaatccatggcggccgc |
| F for 1284 | gagagctagcggcacgtac |
| R for 1284 | cgaaatgatcgcggccatg |
| F for 3436 | gacgagatcgccaagcgtc |
| R for 3436 | gtccggatccgctcaactg |
| F for 5634 | gctgcttgaagcggttgag |
| R for 5634 | gcgtgtggagatagctggtc |
| F for 0582 | accatcgttgcgatcctctc |
| R for 0582 | ctcgactatctcgtcgccac |
| F for 0627 | cgacacgaccgaggatctc |
| R for 0627 | gtcgacgatcgaggacctg |
| F for 2530 | atgagatggcagtcggtgac |
| R for 2530 | ccgtgagtacgtgcagatcg |

**Supplementary Table 3. Codon-optimized DNA sequence of vector components for expression in mycobacteria**

| **>APOBEC1** |  |
| --- | --- |
| ATGTCGTCGGAAACCGGCCCGGTCGCGGTGGACCCGACTTTACGCCGGCGGATCGAGCCGCACGAGTTCGAGGTCTTCTTCGACCCGCGCGAGCTGCGGAAGGAGACTTGTTTACTGTACGAGATCAACTGGGGCGGCCGCCACTCGATCTGGCGCCACACCTCGCAGAACACCAATAAGCACGTCGAGGTCAACTTCATCGAGAAGTTCACCACGGAGCGCTACTTCTGCCCCAACACCCGCTGCTCGATCACTTGGTTTTTAAGCTGGTCGCCGTGCGGGGAGTGCTCGCGGGCCATCACCGAGTTTTTATCGCGCTACCCGCACGTGACTTTATTCATCTACATCGCCCGGCTGTATCACCACGCCGACCCCCGCAACCGGCAAGGTTTGCGCGATTTAATCTCGTCGGGGGTGACCATCCAGATCATGACCGAGCAAGAGTCGGGCTACTGCTGGCGCAACTTCGTCAACTACTCGCCGTCGAACGAGGCCCACTGGCCGCGCTATCCGCATCTGTGGGTCCGGTTGTACGTGCTGGAGTTGTACTGCATCATTTTAGGCTTGCCGCCGTGTTTAAACATTTTACGCCGCAAGCAGCCGCAGCTGACCTTCTTCACCATCGCTTTACAGTCGTGTCACTACCAGCGTTTACCGCCGCACATTTTATGGGCCACCGGCTTGAAGTGA |  |
| **>UGI** |  |
| ATGACCAATTTATCGGACATTATCGAGAAGGAGACCGGCAAGCAACTGGTGATCCAAGAATCGATTTTAATGCTGCCGGAGGAGGTGGAGGAGGTGATCGGCAACAAGCCGGAGTCGGACATTTTAGTGCACACCGCCTACGACGAGTCGACCGACGAGAACGTGATGCTGCTGACCTCGGACGCCCCGGAATACAAGCCGTGGGCGCTGGTGATCCAAGACTCGAACGGCGAGAACAAGATCAAGATGCTGTGA |  |
